# Platelet‐derived extracellular vesicles are increased in sera of Alzheimer's disease patients, as revealed by Tim4‐based assays

**DOI:** 10.1002/2211-5463.13068

**Published:** 2021-02-03

**Authors:** Haruki Odaka, Keiko Hiemori, Asako Shimoda, Kazunari Akiyoshi, Hiroaki Tateno

**Affiliations:** ^1^ Cellular and Molecular Biotechnology Research Institute National Institute of Advanced Industrial Science and Technology Tsukuba Japan; ^2^ Department of Polymer Chemistry Graduate School of Engineering Kyoto University Japan

**Keywords:** Alzheimer’s disease, extracellular vesicles, lectin microarray, platelet, sandwich assay, Tim4

## Abstract

Alzheimer's disease (AD) is the most common form of dementia, characterized by the accumulation of β‐amyloid plaques and the formation of neurofibrillary tangles. Extracellular vesicles (EVs) are small vesicles surrounded by a lipid bilayer membrane, which may be involved in the progression of AD. Glycans are essential building blocks of EVs, and we hypothesized that EV glycans may reflect pathological conditions of various diseases. Here, we performed glycan profiling of EVs prepared from sera of three AD patients (APs) compared to three healthy donors (HDs) using lectin microarray. Distinct glycan profiles were observed. Mannose‐binding lectins exhibited significantly higher signals for AP‐derived EVs than HD‐derived EVs. Lectin blotting using mannose‐binding lectin (rPALa) showed a single protein band at ~ 80 kDa exclusively in AP‐derived EVs. LC‐MS/MS analysis identified a protein band precipitated by rPALa as CD61, a marker of platelet‐derived exosomes (P‐Exo). Sandwich assays using Tim4 with specificity for phosphatidylserine on EVs and antibodies against P‐Exo markers (CD61, CD41, CD63, and CD9) revealed that P‐Exo is significantly elevated in sera of APs (*n* = 16) relative to age‐ and sex‐matched HDs (*n* = 16). Tim4‐αCD63 showed the highest value for the area under the curve (0.957) for discriminating APs from HDs, which should lead to a better understanding of AD pathology and may facilitate the development of a novel diagnostic method for AD.

## Graphical abstract

Glycan profiling and LC‐MS/MS analysis revealed upregulation of exosomes containing a platelet marker CD61 in sera of Alzheimer’s patients (APs) relative to healthy donors (HDs). Quantitative analysis using Tim4‐based sandwich assays provided further evidence that platelet‐derived exosomes (P‐Exo) are increased in sera of APs. Sandwich assays to detect P‐Exo showed high specificity and sensitivity to discriminate APs from HDs.

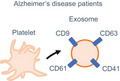

**Abbreviations**
AD: Alzheimer's disease
AP: Alzheimer's disease patient
AUC: area under the curve
Aβ: β‐amyloid
EV: extracellular vesicle
HD: healthy donor
MMSE: Mini‐Mental State Examination
NTA: nano tracking analysis
pAb: polyclonal antibody
P‐Exo: platelet‐derived exosome
P‐MV: platelet‐derived microvesicle
PSM: propensity score matching
ROC: receiving operator curve
TEM: transmission electron microscopy

Alzheimer's disease (AD), the most prevalent cause of dementia, is a growing global health concern with enormous implications for individuals and society [1]. AD is a chronic progressive neurodegenerative disorder which is characterized by aggregation of β‐amyloid (Aβ) plaques and formation of neurofibrillary tangles (NFTs), derived from hyperphosphorylated tau proteins. These factors lead to increasing neural dysfunction. AD is most common cause of dementia in the elderly. Up to 26.6 million people worldwide suffer from AD, and its prevalence is estimated to quadruple by 2050 [2].

Extracellular vesicles (EVs) are small vesicles surrounded by a lipid bilayer membrane. EVs include exosomes of endocytic origin, microvesicles generated by plasma membrane budding, and apoptotic bodies [3]. EVs are important mediators of intercellular communication, as vehicles for proteins, lipids, and nucleic acids during transmission of biological signals between cells [4]. EVs may be involved in progression of AD and nerve cell injury [5]. In contrast, EVs might act on Aβ to reduce injury in the central nervous system [6]. MicroRNA in EVs is also attractive targets as biomarkers for diagnosis of AD [7]. Biomarkers are necessary for improving diagnostic sensitivity and specificity, and for monitoring biological characteristics of AD before manifestation clinical symptoms.

Glycans are also essential building blocks of EVs. We previously analyzed EVs derived from human induced pluripotent stem cells (hiPSCs) for comparison with EVs from non‐hiPSCs using a glycan profiling technology, called high‐density lectin microarray [8]. EVs derived from hiPSCs retain the characteristic glycan signature of hiPSCs. Thus, we hypothesized that glycans of EVs could reflect pathological conditions of various diseases.

In this study, we initially analyzed glycans of EVs isolated from sera of three AD patients (APs) and compared them with EVs from three healthy donors (HDs) using high‐density lectin microarray [9, 10]. AP‐derived EVs showed distinct glycan profiles from HD‐derived EVs. Mannose‐binding lectins showed higher binding to AP‐derived EVs than to HD‐derived EVs. A single protein band at ~ 80 kDa was exclusively detected by mannose‐binding lectin (rPALa) in AP‐derived EVs. The rPALa‐positive protein band was identified as integrin β3, also known as CD61, that is specifically expressed on platelets. Western blotting indicated that αCD61 showed stronger binding to AP‐derived EVs than HD‐derived EVs. Further, antibodies against exosome markers (CD9 and CD63) also showed stronger binding in western blotting to AP‐derived EVs than HD‐derived EVs. Quantitative analysis using Tim4 with specificity for phosphatidylserine (PS) on EVs and antibodies against markers of platelet‐derived exosomes (P‐Exo) demonstrated that P‐Exo is increased in the sera of APs. Overall, we demonstrate for the first time that P‐Exo is increased in sera of APs, which should lead to a better understanding of the pathology of the disease and may allow the development of a novel diagnostic marker for AD.

## Materials and methods

### Study population

Sera from 29 HDs and 20 APs were obtained from BioIVT, which are collected following the written informed consent (Westbury, NY, USA). Participant's demographic data are presented in Table S1. In the quantitative analysis using Tim4‐based sandwich assays, 29 HDs and 20 APs populations were examined as whole cohort. In addition, to correct the difference in age and sex between two groups, we performed 1 : 1 propensity score‐matching (PSM) analysis. After PSM, 16 subjects from each group were selected and examined as PSM cohort. All experiments were designed and performed in accordance with the standards set by the Declaration of Helsinki and guidelines of the National Institute of Advanced Industrial Science and Technology (AIST) ethics committee assigning authors.

### Preparation of EVs

Extracellular vesicles were prepared from serum using MagCapture™ Exosome Isolation Kit PS (Wako Pure Chemical Industries, Ltd., Osaka, Japan) following the manufacturer's instructions [11]. Serum (1 mL) was pre‐cleaned by sequential centrifugation at 1200 ***g*** for 20 min and 10 000 ***g*** for 30 min to remove cells, cellular debris, and large EVs. Supernatants were incubated with Tim4‐immobilized magnetic beads and 1/500 volume of exosome binding enhancer at 4 °C overnight. Subsequently, magnetic beads were washed with washing buffer (1 mL, supplemented with 1/500 volume of exosome binding enhancer) three times. EVs were then eluted with exosome elution buffer (50 µL). Elution step was repeated twice, and totally, 100 µL of EV samples was collected. Protein concentration was quantified with the bicinchoninic acid assay (Thermo Fisher Scientific K.K., Tokyo, Japan). The size distribution and particle number of the prepared EVs were analyzed by nanoparticle tracking analysis (NTA). EVs were diluted to a concentration of 4–8 × 10^8^ particles per mL with PBS and analyzed in triplicates using a Nanosight LM10 system (Marvern Instruments Ltd., Worcestershire, UK) equipped with a blue laser. Experimental conditions were as follows: Measurement Time: 60 s; Blur: Auto; Detection Threshold: 4–5; Min Track Length: Auto; Min Expected Size: Auto. Representative data are shown of three independent experiments. Morphology of EVs was examined using an HT7700‐transmission electron microscopy (TEM). EVs (5 µL) were mixed with 4% paraformaldehyde (5 µL), incubated with Formvar film for TEM (PVF‐C10 STEM; Okenshoji Co., Ltd., Tokyo, Japan) for 10 min, and negatively stained with 2% phosphotungstic acid for 20 s. After dry, EVs were observed by TEM. Representative data are shown.

### Lectin microarray production

Ninety‐six lectins (Table S2) were dissolved at a concentration of 0.5 mg·mL^−1^ in a spotting solution (Matsunami Glass, Osaka, Japan), and spotted onto epoxysilane‐coated glass slides (SCHOTT Japan Corporation, Tokyo, Japan) in triplicate using a non‐contact microarray printing robot (MicroSys4000; Fordx, Tokyo, Japan) [9, 10]. The glass slides were then incubated at 25 °C overnight to allow lectin immobilization. The lectin‐immobilized glass slides were washed with probing buffer (25 mm Tris/HCl, pH 7.5, 140 mm NaCl (TBS) containing 2.7 mm KCl, 1 mm CaCl_2_, 1 mm MnCl_2_, and 1% Triton X‐100) and incubated with blocking reagent N102 (NOF Co., Tokyo, Japan) at 20 °C for 1 h. Finally, the lectin‐immobilized glass slides were washed with TBS containing 0.02% NaN_3_ and stored at 4 °C until use. The spot quality and reproducibility of the produced microarrays were checked before use, using a Cy3‐labeled test probe containing 250 μg·mL^−1^ asialofetuin (Sigma‐Aldrich Japan, Tokyo, Japan), 25 ng·mL^−1^ Siaα2–3Galβ1–4GlcNAc‐BSA (Dextra Laboratories Ltd., Reading, UK), 10 ng·mL^−1^ Fucα1–2Galβ1–3GlcNAcβ1–3Galβ1–4Glc‐BSA (Dextra Laboratories Ltd.), 10 ng·mL^−1^ βGlcNAc‐BSA (Dextra Laboratories Ltd.), 10 ng·mL^−1^ GalNAcα1–3(Fucα1–2)Gal‐BSA (Dextra Laboratories Ltd.), 10 ng·mL^−1^ Galα1–3Galβ1‐4GlcNAc‐BSA (Dextra Laboratories Ltd.), 10 ng·mL^−1^ Manα1–3(Manα1–6)Man‐BSA (Dextra Laboratories Ltd.), 10 ng·mL^−1^ αFuc‐BSA (Dextra Laboratories Ltd.), 10 ng·mL^−1^, αGalNAc‐BSA (Dextra Laboratories Ltd.), and 10 ng·mL^−1^ Siaα2–6Galβ1–4Glc‐BSA (Dextra Laboratories Ltd.) dissolved in probing buffer.

### Lectin microarray analysis

Extracellular vesicles (0.4 µg) were labeled with Cy3‐*N*‐hydroxysuccinimide ester (GE Healthcare Ltd., Tokyo, Japan), diluted with probing buffer [25 mm Tris/HCl (pH 7.5), 140 mm NaCl, 2.7 mm KCl, 1 mm CaCl_2_, 1 mm MnCl_2_, and 1% Triton X‐100] to 0.5 µg·mL^−1^, and incubated in high‐density lectin microarray at 20 °C overnight. After washing with probing buffer, fluorescence images were captured using a Bio‐Rex scan 200 evanescent‐field activated fluorescence scanner (Rexxam Co. Ltd., Kagawa, Japan). Lectin signals of triplicate spots were averaged for each protein sample and normalized relative to the mean value of all 96 lectins. An unpaired Student's *t*‐test was performed for comparison of the lectin signal in two groups. Having derived *P*‐value for each lectin, *q*‐values were calculated using the Storey approach and statistical significance was set at *q*‐value < 0.05 [12]. Unsupervised clustering was performed by employing the average linkage method using cluster 3.0 software [13]. The heat map with clustering was acquired using Java Treeview. Principal component analysis (PCA) was performed by spss statistics 19 (IBM Japan, Tokyo, Japan).

### SDS/PAGE and western blotting

Equal amount of proteins or particle numbers of serum‐derived EVs were electrophoresed under non‐reducing conditions on 5–20% polyacrylamide gels (DRC, Tokyo, Japan) and visualized using a Silver Staining MS kit (Wako Pure Chemical Industries, Ltd) following the manufacturer's instructions. Separated proteins were transferred to a poly(vinylidene difluoride) membrane. After blocking with BlockAce (Funakoshi Co., Ltd., Tokyo, Japan), membranes were incubated with αCD61 polyclonal antibody (pAb; 1 µg·mL; R&D Systems, Inc., Minneapolis, MN, USA), αCD41 pAb (2 µg·mL; R&D Systems, Inc), αCD51 pAb (1 µg·mL^−1^; R&D Systems, Inc.), αCD9 monoclonal antibody (mAb) (×500, clone No.: Ts9; Thermo Fisher Scientific K.K.), or αCD63 mAb (×2000, clone No.: 8A12; COSMO BIO Co. Ltd., Tokyo, Japan), followed by horseradish peroxidase (HRP)‐conjugated AffiniPure donkey αgoat IgG (H + L) (×10  000; Jackson ImmunoResearch Inc., West Grove, PA, USA) or HRP‐labeled AffiniPure goat αmouse IgG (H + L) (×10 000; Jackson ImmunoResearch Inc.).

### Lectin‐precipitation and LC‐MS/MS

Biotinylated rPALa lectin was immobilized on Dynabeads M280 Streptavidin (Thermo Fisher Scientific K.K.) and incubated with protein samples extracted from EVs at 4 °C overnight. After washing with PBST (PBS containing 1% Triton X‐100), bound proteins were eluted with 0.2% SDS solution at 95 °C for 5 min and cooled on ice. The elution fraction was separated by SDS/PAGE followed by silver staining as described above. Protein bands were diced into small pieces, washed with diluted water and acetonitrile, and dried under reduced pressure. Subsequently, gel pieces were incubated with trypsin at 37 °C for 16 h. Tryptic peptides were extracted with 5% formic acid and 50% acetonitrile buffer containing 0.1% trifluoroacetic acid, followed by desalting with C18 spin columns and dried under reduced pressure. Dried peptides were diluted in 3% acetonitrile and 0.1% formic acid, and analyzed by nano LC‐MS/MS system composed of Q Exactive HF‐X (Thermo Fisher Scientific K.K.) and UltiMate 3000 RSLCnano LC System (Thermo Fisher Scientific K.K.). Acquired MS/MS data were searched using PEAKS Studio X+ (Bioinformatics Solutions Inc., Waterloo, Canada) against amino acid sequences in the reviewed UniProtKB/Swiss‐Prot database (*Homo sapiens*). Search parameters were as follows: Digestion Enzyme, Trypsin; Precursor tolerance, 10 p.p.m.; Fragment tolerance, 0.015 Da; Maximum missed cleavage, 2; Variable Modifications, Oxidation (M). Peptide matching criteria were set at the number of unique peptides per protein ≥ 2. We excluded the desmosomal proteins including keratin family, desmoglein, and plakoglobin, from the analysis, since these proteins were potentially originated from the contaminated human skin and hair [14]. Glycoproteins were determined by searching in the UniProt database (https://www.uniprot.org/).

### Quantification of EVs

P‐Exo marker (CD41, CD61, CD9, CD81, and CD63)‐positive EVs in sera were quantified using PS Capture Exosome ELISA Kit (Streptavidin HRP) (Wako Pure Chemical Industries, Ltd) following the manufacture's protocol with modifications. Tim4‐coated plates were incubated with 100 µL of sera (10‐fold dilution: Tim4‐αCD81, 100‐fold dilution: Tim4‐αCD9, Tim4‐αCD63, Tim4‐αCD41, Tim4‐αCD61) for 2 h at room temperature (RT). After washing, biotinylated antibody for each P‐Exo marker was incubated for 1 h at RT. HRP‐labeled streptavidin was then added and incubated for 2 h at RT. After washing, TMB solutions were added. After 30 min, stop solution was applied, and optical density (OD) was measured at 450 nm as the dominant wavelength and 620 nm as the secondary wavelength. The following antibodies were biotinylated and used for sandwich assays: αCD61 mAb (1 µg·mL^−1^, clone No.: 256809; R&D Systems), αCD41 mAb (1 µg·mL^−1^, clone No.: 745201; R&D Systems), αCD9 mAb (0.1 µg·mL^−1^, clone No.: Ts9; Thermo Fisher Scientific K.K.), and αCD81 (0.1 µg·mL^−1^, SHI‐EXO‐M03; Cosmo Bio Co., Ltd.).

### Statistical analysis

Wilcoxon–Mann–Whitney test, Chi‐squared test, Spearman's rank correlation test, PSM analysis, and receiving operator curve (ROC) analysis were performed using EZR on R commander version 1.41 [15]. Statistical significance was set at *P*‐value < 0.05.

## Results

### Glycan profiling of EVs derived from sera of APs

**Fig. 1:**
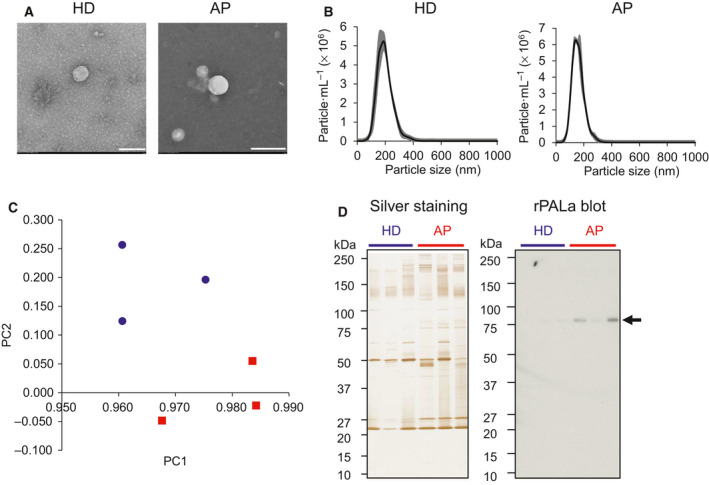
Glycan profiling of EVs derived from sera of HDs and APs. (A) Representative TEM images of EVs isolated from sera of HDs and APs. Scale bar: 200 nm. (B) Size distribution of HD‐ and AP‐derived EVs analyzed by NTA (*n* = 3). Gray area indicates SD. (C) PCA plot of lectin microarray data of HD (blue)‐ and AP (red)‐derived EVs. Sample No. 4, 5, 6 for HDs and No. 30, 31, 32 for APs were used (Table S1). *X*‐ and *Y*‐axis shows the first and second principal component (PC), respectively. (D) Representative images of silver staining and lectin blotting using mannose‐binder (rPALa) of HD and AP‐derived EVs separated by SDS/PAGE. Arrow indicates rPALa‐positive 80kDa proteins. Sample No. 4, 5, 6 for HDs and No. 30, 31, 32 for APs were used (Table S1).

In order to isolate small EVs, we used Tim4‐affinity method, which has been reported to isolate EVs with high purity and low protein contaminants [11]. EVs were captured from sera of APs and HDs using magnetic beads immobilized with mouse Tim4, which binds specifically to PS exposed on EVs in a Ca^2+^‐dependent manner [11]. Lipid bilayer vesicles in purified EV fractions from APs and HDs were examined by TEM (Fig. 1A). NTA revealed that average diameters of AP‐derived EVs and HD‐derived EVs were 197 ± 10.2 and 159 ± 1.11 nm (average ± SD), respectively (Fig. 1B). The number of particles at 1 µg·mL^−1^ was 6 × 10^8^ (AP‐derived EVs) and 3 × 10^8^ (HD‐derived EVs) particles per mL. Small EVs could be purified from sera of both APs and HDs.

The purified EVs were fluorescently labeled with Cy3‐N‐hydroxysuccinimide ester and incubated in a high‐density lectin microarray containing 96 lectins (Table S2). Fluorescence signals were normalized by the average signals of 96 lectins and assessed by principal component and cluster analysis (Figs 1C and S1). EVs derived from APs and HDs were clearly separated. By Student's *t*‐test, 13 lectins were selected that show significantly different signals between AP‐ and HD‐derived EVs (*q* < 0.05, Table 1). Importantly, 8 of 13 lectins showed binding specificity to mannosylated glycans, suggesting higher expression of mannosylated glycans in AP‐derived EVs than HD‐derived EVs (Table 1).

**Table 1:** Significantly different lectins between HD‐ and AP‐derived EVs. Gal, D‐galactose; GlcNAc, N‐acetyl‐glucosamine; Sia, sialic acid; Man, mannose; biantenna, biantennary N‐glycans; triantenna, triantennary N‐glycans.

Lectin	*t*‐value	*P*‐value^a^	*q*‐value^b^	Rough specificity^c^
HHL	−6.4884	0.0029	0.0434	**Manα1‐3Man, Manα1‐6Man**
VVAII	−6.4882	0.0029	0.0434	**Man**, agalacto
ConA	−6.3758	0.0031	0.0434	**M3, Manα1‐2Manα1‐3(Manα1‐6)Man, GlcNAcβ1‐2Manα1‐3(Manα1‐6)Man**
ASA	−6.2292	0.0034	0.0434	Galβ1‐4GlcNAcβ1‐2Man
LEL	5.9212	0.0041	0.0434	Polylactosamine (GlcNAc)_n_
ECA	−5.5052	0.0053	0.0434	βGal
rPALa	−5.3548	0.0059	0.0434	**Man5**, biantenna
GNA	−5.2205	0.0064	0.0434	**Manα1‐3Man, Manα1‐6Man**
rPSL1a	4.8653	0.0082	0.0443	α2‐6Sia
rGRFT	−4.7914	0.0087	0.0443	**Man**
rBanana	−4.7266	0.0091	0.0443	**Manα1‐2Manα1‐3(6)Man**
rOrysata	−4.6243	0.0098	0.0443	**Manα1‐3Man, Highman**, biantenna
CCA	−4.5109	0.0107	0.0446	Galactosylated N‐glycans up to triantenna

^a^ *P*‐value in unpaired, two‐tailed *t*‐test.

^b^ *q*‐value was calculated using the Storey approach.

^c^ Mannosylated glycans were shown in bold style.

### Lectin blotting and identification of carrier proteins

We searched for carrier proteins of mannosylated glycans in AP‐derived EVs. Lectin blotting using mannose‐binding lectin (rPALa) showed a single protein band at 80 kDa in EVs purified from sera of patients of AD, but not HD‐derived EVs (Fig. 1D). In order to identify the 80 kDa protein, protein lysates of AP‐ and HD‐derived EVs were incubated with rPALa‐immobilized beads followed by SDS/PAGE and silver staining. The protein band at 80 kDa was then analyzed by LC‐MS/MS. Eleven glycoproteins with higher counts in AP than HD were detected by LC‐MS/MS (Table S3). Among them, integrin β3, also known as CD61 and GPIIIa, was the sole protein with the average molecular weight around 80 kDa [16]. Higher count of integrin β3 was detected in AP‐derived EVs (spectral count: 9) than HD‐derived EVs (spectral count: 13) (Table S3). CD61 is a glycoprotein containing six N‐glycosylation sites, specifically expressed on platelets and megakaryocytes [17].

### Qualitative analysis by western blotting

**Fig. 2:**
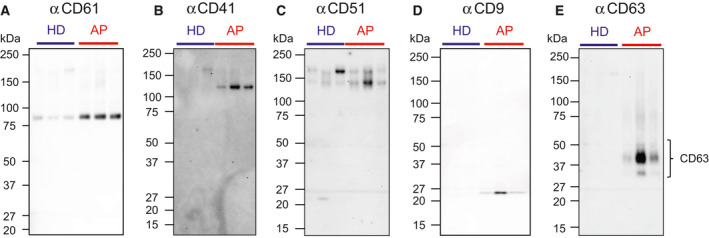
Western blotting of HD‐ and AP‐derived EVs. Purified HD‐ and AP‐derived EVs (3.6 × 10^7^ particle / lane) were separated on SDS/PAGE and blotted with αCD61 (A), αCD41 (B), αCD51 (C), αCD9 (D) and αCD63 (E). Lane 1: No. 12, lane 2: No. 18, lane 3: No. 19, lane 4: No. 40, lane 5: No. 43, lane 6: No. 49 (Table S1).

In order to confirm whether CD61 shows higher expression in AP‐derived EVs than HD‐derived EVs, we then performed western blotting to confirm the results of Fig. 1. The equal number of particles or the equal protein amounts were run on SDS/PAGE and blotted with antibodies (Figs 2 and S2). Consistently, αCD61 showed stronger staining to AP‐derived EVs than HD‐derived EVs at 80 kDa in western blots (Figs 2 and S2). CD61 forms heterodimeric complexes with CD41 (integrin αII) as well as CD51 (integrin αV). CD61/CD41 complex is known to be expressed exclusively on platelets and megakaryocytes, whereas CD61/CD51 complex is expressed on various cell types such as endothelium, fibroblasts, smooth muscle, osteoclasts, and leukocytes [18]. Anti‐CD41 pAb showed higher intensity to AP‐derived EVs than HD‐derived EVs (Fig. 2B). In contrast, no obvious difference of αCD51 blot was observed between HD‐ and AP‐derived EVs (Fig. 2C). Thus, we hypothesized that platelet‐derived EVs (P‐EVs) were enriched in AP‐derived EVs. Heijnen *et al*. [19] classified P‐EVs into two types: exosomes (P‐Exos) and microvesicles (P‐MVs). P‐Exos are enriched in CD9, CD63, and CD41/CD61, whereas factor X and prothrombin are restricted to P‐MVs [20]. Antibodies against conventional exosome markers, CD9 and CD63, also showed greater binding to AP‐derived EVs than HD‐derived EVs (Figs 2D,E and Fig. S2). αCD63 showed broad protein bands ranging from 30 to 50 kDa, since CD63 is glycosylated [21]. Consistently, the 27 kDa protein band corresponding to CD9 was much stronger in AP‐derived EVs than HD‐derived EVs revealed in silver staining (Fig. 1D). These results suggest that P‐Exos positive with CD9, CD63, CD41, and CD61, might be increased in APs relative to HDs.

### Quantitative analysis by Tim4‐based sandwich assays

**Fig. 3:**
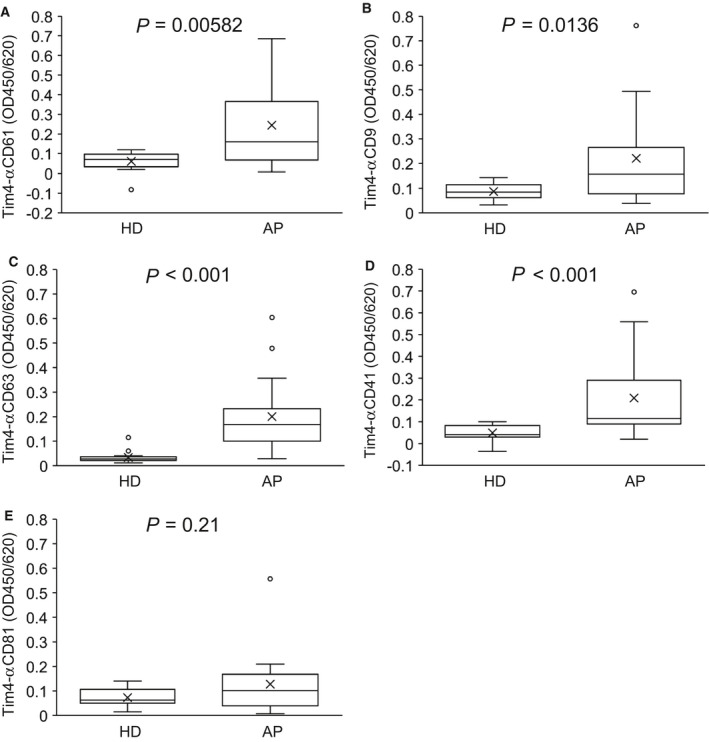
Quantitative analysis of HD‐ and AP‐derived EVs using Tim4‐based sandwich assays in PSM cohort. Box‐whisker plots of the data of HDs (*n* = 16) and APs (*n* = 16) analyzed by sandwich assays using immobilized Tim4 and overlay antibodies against CD61 (A), CD9 (B), CD63 (C), CD41 (D), and CD81 (E). *P*‐values obtained by Wilcoxon–Mann–Whitney test are indicated in the figure.

The increase of P‐Exos in AP‐derived EVs was validated and quantified with sandwich assays using Tim4‐immobilized microplates and antibodies against CD61, CD41, CD9, and CD63. Antibody against another exosome marker, CD81, was also used as a detection probe. Five sandwich assays were constructed and applied for the analysis of sera of 29 HDs and 20 APs. Tim4‐coated microplates were incubated directly with sera of HDs and APs, and detected with antibodies against CD61, CD9, CD63, CD41, and CD81. Participant's demographic data are presented in Table S1. Consistent with western blotting data, values of Tim4‐αCD61, Tim4‐αCD9, Tim4‐αCD63, and Tim4‐αCD41 were significantly elevated in AP‐derived sera (Fig. S3a‐d). Tim4‐αCD81 sandwich assay showed only a slight increment for AP sera (Fig. S3e). We found that there is a significant difference in age between two groups in whole cohort, indicating that the effect of age cannot be ignored (Table 2). Indeed, values of Tim4‐αCD9 and Tim4‐αCD81 sandwich assays showed a weak but significant correlation with age (*P* < 0.05) in the HD group (Table S4). Therefore, we performed 1 : 1 propensity score matching (PSM) to correct the difference in age and sex. After PSM, 16 samples were selected in each group, with no significant differences in age and sex (Table 2). Even in PSM cohort, the values of Tim4‐αCD61, Tim4‐αCD41, Tim4‐αCD9, and Tim4‐αCD63 were significantly higher in the AP group (Fig. 3A‐D). In contrast, Tim4‐αCD81 showed comparable signals between APs and HDs (Fig. 3E). The value of Tim4‐αCD41 was highly correlated with Tim4‐αCD61 (ρ = 0.914). Interestingly, values of Tim4‐αCD41 and Tim4‐αCD61 showed high correlation with Tim4‐αCD9 (ρ > 0.7), moderate with Tim4‐αCD63 (ρ > 0.6), but low with Tim4‐αCD81 (ρ < 0.3; Table S5).

**Table 2:** Participant's characteristics of demographic data before and after PSM.

	Whole cohort			PSM cohort		
	HD	AP	*P‐*value^a^	HD	AP	*P‐*value
*N*	29	20		16	16	
Sex (male/female)	19/10	14/6	1	9/7	12/4	0.458
Age (year)	34.00 [18.00, 69.00]^b^	62.00 [48.00, 84.00]	<0.001	60.00 [33.00, 69.00]	58.00 [48.00, 72.00]	0.396

^a^ *P*‐value in Chi‐Squared Test (sex) or Wilcoxon‐Mann‐Whitney test (age).

^b^ 95% confidence interval.

### Correlation analysis between the values of Tim4‐based sandwich assays and MMSE score

**Fig. 4:**
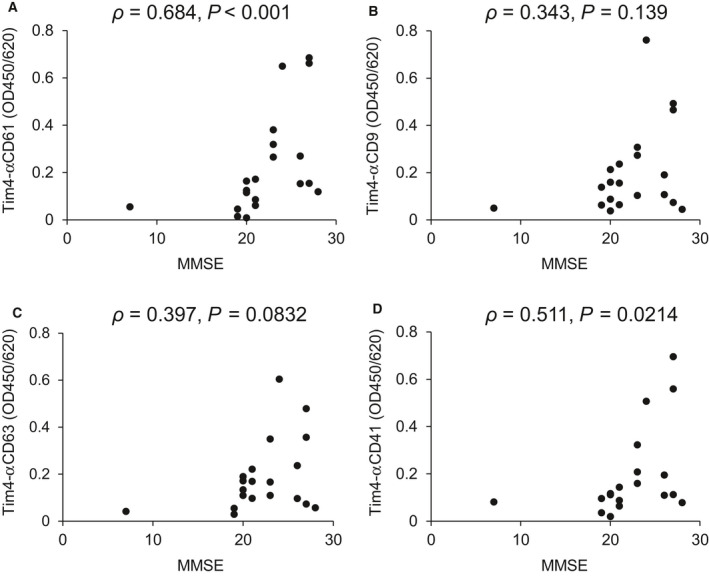
Correlation between the values obtained by Tim4‐based sandwich assays with MMSE scores. Correlation diagrams between MMSE score and OD values of the sandwich assays using Tim4 and antibodies against CD61 (A), CD9 (B), CD63 (C), and CD41 (D) in APs (*n* = 20). *Y*‐axis shows OD450/620 determined by sandwich assays. The *X*‐axis shows MMSE scores of APs. Correlation was examined by Spearman's rank correlation coefficient analysis, and *P*‐values are indicated in the figure.

Mini‐Mental State Examination (MMSE) is widely used for screening of cognitive impairment. APs can experience a decline of two points or less per year on their MMSE score [22]. Possible correlations between the values of Tim4‐based sandwich assays and MMSE score in APs were examined. Both Tim4‐αCD41 (ρ = 0.511, *P* = 0.0214) and Tim4‐αCD61 (ρ = 0.684, *P* < 0.001) were positively correlated with MMSE score, suggesting that CD41‐ and CD61‐positive EVs are elevated in the early stages of AD relative to late‐stage AD. In contrast, Tim4‐αCD63 (ρ = 0.397, *P* = 0.0832) and Tim4‐αCD9 (ρ = 0.343, *P* = 0.139) showed no significant correlation with MMSE scores (Fig. 4A‐D). No correlation between age and MMSE score in the AP group was observed (ρ = 0.169, *P* = 0.477).

### ROC curve analysis of Tim4‐based sandwich assays

**Fig. 5:**
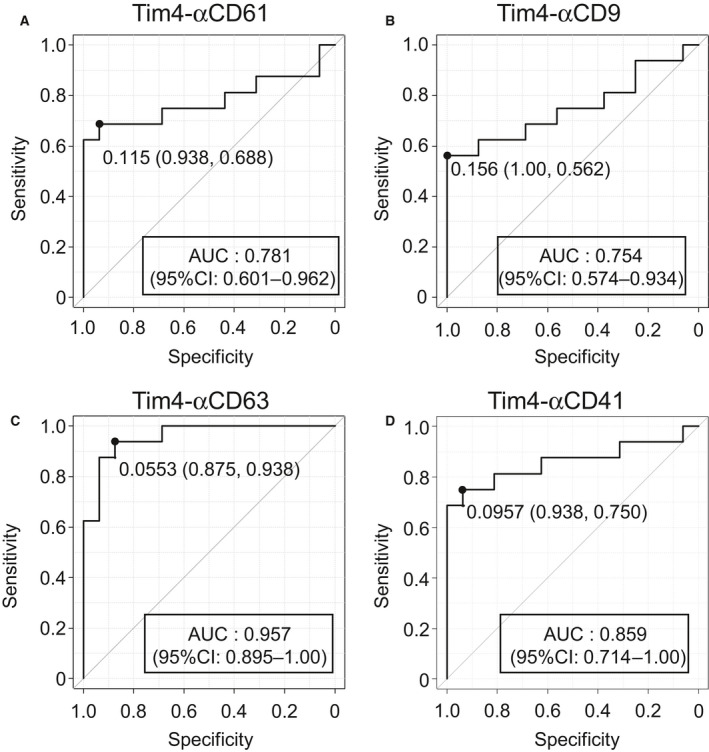
ROC curves of the values obtained by Tim4‐based sandwich assays for predicting the risk of AD. ROC curves for predicting AD risk by OD values (HDs: *n* = 16, APs: *n* = 16) obtained by sandwich assays using Tim4 and antibodies against CD61 (A), CD9 (B), CD63 (C), and CD41(D). AUC and its 95% confidence interval (CI) are indicated in the figure. ‘Sensitivity’ indicates true positive rate and ‘Specificity’ indicates (1—false positive rate).

Receiver Operating Characteristic (ROC) curves were generated to further evaluate the predictive value of Tim4‐based sandwich assays for AD risk in whole and PSM cohort (Figs 5 and S4). In PSM cohort, area under the ROC curves (AUC) were 0.781 for Tim4‐αCD61 (95% CI: 0.601–0.962), 0.754 for Tim4‐αCD9 (95% CI: 0.574–0.934), 0.957 for Tim4‐αCD63 (95% CI: 0.895–1.000) and 0.859 for Tim4‐αCD41 (95% CI: 0.714–1.00; Fig. 5A‐D). The thresholds that maximize the sum of sensitivity and specificity were 0.115 (specificity: 0.938, sensitivity: 0.688) for Tim4‐αCD61, 0.156 (specificity: 1.00, sensitivity: 0.562) for Tim4‐αCD9, 0.0553 (specificity: 0.875, sensitivity: 0.938) for Tim4‐αCD63, and 0.0957 (specificity: 0.938, sensitivity: 0.750) for Tim4‐αCD41 (Fig. 5A‐D). Among the developed sandwich assays, Tim4‐αCD63 provided the highest AUC (0.957) to predict AD risk.

## Discussion

In the last two decades, biological fluid‐derived EVs have been intensively investigated as biomarkers for diagnosis and therapy of various diseases, such as cancer, cardiovascular disease, and neurodegenerative disease [23, 24, 25]. We performed glycan profiling of EVs in sera of APs using high‐density lectin microarray. Mannose‐binding lectins were enriched and displayed increased signals in APs compared to HDs. The mannose‐binding lectin rPALa showed a single specific band at 80 kDa, which was identified as CD61. Since membrane proteins are typically modified with complex N‐glycans, mannosylation of CD61 might reflect unique functions for EVs, such as cellular uptake and tissue specificity.

P‐EVs were first discovered in 1967 as ‘platelet dusts’, tiny membrane fragments, released from platelets with the same procoagulant activity as platelets [26]. P‐EVs ranged from 30 to 1000 nm in diameter and are characterized by expression of platelet markers (e.g., CD41, CD61) [20]. P‐EVs are reported to be produced from platelets upon activation by various stimuli, including exposure to platelet agonists (thrombin and collagen), complement proteins, shear stress, and adhesion to vessel walls [27]. P‐EVs are categorized into two groups, P‐Exos and P‐MVs. We demonstrate that P‐Exos, relatively small P‐EVs, ranging from 30 to 200 nm in diameter, are increased in sera of APs. Several lines of evidence show increased platelet activation in APs [28]. Sevush *et al*. [29] showed increased levels of platelet aggregates, expression of CD62p on platelets, and leukocyte‐platelet complexes in AP groups. Ciabattori *et al*. reported increased levels of urinary 11‐dehydro‐thromboxane B_2_ (platelet activation marker), 8‐iso‐prostaglandin (lipid oxidation marker), and decreased vitamin E (antioxidant) in APs, suggesting that the platelet activation is accompanied by oxidative stress and a decrease in antioxidants [30]. Bermejo *et al*. [31] showed augmented platelet levels of COX2 and plasma IL‐6 level in AP groups, suggesting that activation of platelets with peripheral inflammatory responses. Prodan *et al* demonstrated that coated platelets, which are collagen and thrombin activated platelets, were significantly elevated in APs compared to age‐matched controls. The number of coated platelets is positively correlated with MMSE score in APs [32], similar to our results obtained by Tim4‐αCD41 and Tim4‐αCD61 sandwich assays. Thus, increased levels of P‐Exos in sera of APs might be due to platelet activation.

An increase in circulating P‐EVs has also been reported in a variety of diseases including cancer, infections, rheumatoid arthritis, and stroke [27, 33]. P‐EVs display a variety of bioactive substrates, such as growth factors, cytokines, and lipids that affect the pathophysiology of disease. For example, P‐EVs promote cancer cell proliferation and invasion by stimulating MAPK signaling and increasing matrix metalloproteinases [34]. P‐EVs also enhance the formation of new blood vessels during tumor growth via concerted action of FGF‐2, VEGF, and a lipid factor [27, 35]. P‐EVs could trigger neurogenesis and angiogenesis by stimulating ERK and PI3K/Akt signaling in endogenous neural stem/progenitor cells after a stroke [36]. Conversely, P‐EVs exacerbate inflammation in rheumatoid arthritis through IL‐1‐induced secretion of pro‐inflammatory cytokines in synoviocytes [37]. Although the pathological role of P‐EVs on APs is unclear, these studies suggest that P‐EVs may counteract neurodegeneration in the brain of APs by enhancing angiogenesis and neurogenesis. Alternatively, P‐EVs may exacerbate AD pathology by amplifying inflammation. The pathological role of increased P‐EVs in APs will need to be addressed in future studies.

In this study, we developed Tim4‐based sandwich assays to characterize EVs in sera of APs. Values of Tim4‐αCD61, Tim4‐αCD9, Tim4‐αCD63, and Tim4‐αCD41 were increased in sera from APs, whereas Tim4‐αCD81 showed comparable signals between APs and HDs, suggesting that P‐Exos positive for PS, CD61, CD41, CD9, and CD63, are exposed on exosomes released from platelets. In contrast, CD81 might be expressed on EVs of different origins. Since there is no specific marker for each type of EVs: exosome, microvesicle, apoptotic body, it is difficult to know which types of EVs were specifically detected by Tim4‐based sandwich assays. The Tim4‐αCD63 sandwich assay showed the best performance for predicting AD (AUC: 0.957) in ROC curve analysis, indicating that CD63‐positive EVs are increased in sera of AP. Tim4‐αCD41 and Tim4‐αCD61 might be suitable for specific detection of P‐EVs. However, it should be noted that the elevation of P‐EVs might not be AD‐specific pathology as mentioned above, and Tim4‐αCD63 sandwich assay may also respond to sera of patients with other diseases. Further studies are essential to apply this finding to clinical settings.

## Conflict of interest

The authors declare no conflict of interest.

## Author contributions

HT conceived and designed the project, interpreted the data, and wrote the paper. HO performd experiments, analyzed and interpreted the data, and wrote the paper. KH, AS, and KA performed experiments and analyzed the data.

## Supporting information


**Table S1.** List of sera from HDs and APs.
**Table S2.** Lectins used for lectin microarray.
**Table S3.** Identification of rPALa‐precipitated glycoproteins at 80 kDa by LC‐MS/MS.
**Table S4.** Spearman's rank correlation coefficient between the values of sandwich assays and ages in HDs and APs.
**Table S5.** Spearman's rank correlation coefficient among Tim4‐based sandwich assays.
**Fig. S1.** Cluster analysis of lectin microarray data of EVs purified from sera of HDs and APs. Lectin microarray data of EVs purified from sera of APs (*n* = 3) and HDs (*n* = 3) were normalized, log‐transformed, and analyzed by Cluster 3.0 with average linkage methods. The zero value of lectin signal was converted to 1. Sample No.4, 5, 6 for HDs and No.30, 31, 32 for APs were used in Table S1. yellow: high, blue: low.
**Fig. S2.** Western blotting of HD‐ and AP‐derived EVs. Equal amount of proteins (0.2 μg) of purified HD‐ and AP‐derived EVs were separated on SDS/PAGE and blotted with αCD61 (a), αCD9 (b) and αCD63 (c). Lane 1: sample No. 4, lane 2: No. 5, lane 3: No. 6, lane 4: No. 30, lane 5: No. 31, lane 6: No. 32.
**Fig. S3.** Quantitative analysis of HD‐ and AP‐derived EVs using Tim4‐based sandwich assays in whole cohort. Box‐whisker plots of the data of whole cohort (HDs: *n* = 29, APs: *n* = 20) analyzed by sandwich assays using immobilized Tim4 and overlay antibodies against CD61 (a), CD9 (b), CD63 (c), CD41 (d), and CD81 (e). OD: optical density. Sera used in this study are listed in Table S1. *P*‐values obtained by Wilcoxon‐Mann‐Whitney Test are indicated in the figure.
**Fig. S4.** ROC curves of the values obtained by Tim4‐based sandwich assays in whole cohort. ROC curves for predicting AD risk by OD values obtained by sandwich assays (HDs: *n* = 29, APs: *n* = 20) using Tim4 and antibodies against CD61 (a), CD9 (b), CD63 (c), and CD41 (d). Area‐under‐curve (AUC) and its 95% confidence interval (CI) are indicated in the figure.

## Data Availability

The raw data are available from the corresponding author upon reasonable request.
