# Supplementary material for: Platelet‐derived extracellular vesicles are increased in sera of Alzheimer's disease patients, as revealed by Tim4‐based assays
Source: FEBS Open Bio. 2021 Feb 3;11(3):741–52. doi: 10.1002/2211-5463.13068 (PMC7931225; doi:10.1002/2211-5463.13068)
Supplement: Supplementary file 1 — Table S1. List of sera from HDs and APs. Table S2. Lectins used for lectin microarray. Table S3. Identification of rPALa‐precipitated glycoproteins at 80 kDa by LC‐MS/MS. Table S4. Spearman's rank correlation coefficient between the values of sandwich assays and ages in HDs and APs. Table S5. Spearman's rank correlation coefficient among Tim4‐based sandwich assays. Fig. S1. Cluster analysis of lectin microarray data of EVs purified from sera of HDs and APs. Lectin microarray data of EVs purified from sera of APs (n = 3) and HDs (n = 3) were normalized, log‐transformed, and analyzed by Cluster 3.0 with average linkage methods. The zero value of lectin signal was converted to 1. Sample No.4, 5, 6 for HDs and No.30, 31, 32 for APs were used in Table S1. yellow: high, blue: low. Fig. S2. Western blotting of HD‐ and AP‐derived EVs. Equal amount of proteins (0.2 μg) of purified HD‐ and AP‐derived EVs were separated on SDS/PAGE and blotted with αCD61 (a), αCD9 (b) and αCD63 (c). Lane 1: sample No. 4, lane 2: No. 5, lane 3: No. 6, lane 4: No. 30, lane 5: No. 31, lane 6: No. 32. Fig. S3. Quantitative analysis of HD‐ and AP‐derived EVs using Tim4‐based sandwich assays in whole cohort. Box‐whisker plots of the data of whole cohort (HDs: n = 29, APs: n = 20) analyzed by sandwich assays using immobilized Tim4 and overlay antibodies against CD61 (a), CD9 (b), CD63 (c), CD41 (d), and CD81 (e). OD: optical density. Sera used in this study are listed in Table S1. P‐values obtained by Wilcoxon‐Mann‐Whitney Test are indicated in the figure. Fig. S4. ROC curves of the values obtained by Tim4‐based sandwich assays in whole cohort. ROC curves for predicting AD risk by OD values obtained by sandwich assays (HDs: n = 29, APs: n = 20) using Tim4 and antibodies against CD61 (a), CD9 (b), CD63 (c), and CD41 (d). Area‐under‐curve (AUC) and its 95% confidence interval (CI) are indicated in the figure. [file FEB4-11-741-s001.pdf]

Supplementary Table 1. List of sera from HDs and APs

Sample No.	ID	Condition	Age	Sex	MMSE
1	BRH1435559	HD	26	Male	N/A
2	BRH1435560	HD	27	Male	N/A
3	BRH1435561	HD	34	Male	N/A
4	BRH1435562	HD	18	Male	N/A
5	BRH1435563	HD	24	Male	N/A
6	BRH1435564	HD	27	Male	N/A
7	BRH1435565	HD	33	Male	N/A
8	BRH1435566	HD	34	Male	N/A
9	BRH1435567	HD	29	Male	N/A
10	BRH1435568	HD	33	Male	N/A
11	BRH1435569	HD	28	Female	N/A
12	BRH1435570	HD	33	Female	N/A
13	BRH1435571	HD	44	Female	N/A
14	BRH1435572	HD	26	Female	N/A
15	BRH1435573	HD	37	Female	N/A
16	BRH1435574	HD	36	Female	N/A
17	BRH1435575	HD	39	Female	N/A
18	BRH1435576	HD	32	Female	N/A
19	BRH1435577	HD	18	Female	N/A
20	BRH1435578	HD	28	Female	N/A
21	BRH1452812	HD	66	Male	N/A
22	BRH1452814	HD	61	Male	N/A
23	BRH1452815	HD	66	Male	N/A
24	BRH1452816	HD	69	Male	N/A
25	BRH1452817	HD	68	Male	N/A
26	BRH1452818	HD	60	Male	N/A
27	BRH1452819	HD	60	Male	N/A
28	BRH1452820	HD	67	Male	N/A
29	BRH1452821	HD	60	Male	N/A
30	BRH1441067	AP	56	Male	20
31	BRH1441068	AP	54	Female	20
32	BRH1449536	AP	48	Male	21
33	BRH1449538	AP	63	Male	21
34	BRH1449537	AP	55	Female	21
35	BRH1449520	AP	54	Female	19
36	BRH1449519	AP	65	Male	20
37	BRH1453755	AP	54	Female	19

38	BRH1453754	AP	65	Male	20
39	BRH1461098	AP	69	Male	23
40	HMN75708	AP	52	Male	27
41	HMN75709	AP	72	Male	23
42	HMN75710	AP	75	Female	26
43	HMN75711	AP	60	Male	24
44	HMN75712	AP	84	Male	28
45	HMN75713	AP	64	Male	26
46	HMN75714	AP	68	Female	7
47	HMN75715	AP	83	Male	23
48	HMN79957	AP	61	Male	27
49	HMN79955	AP	51	Male	27

Supplementary Table 2. Lectins used for lectin microarray[1]

	Name	Species	Origin	Source[3]	Rough specificity[2]
1	LFA	*Limax flavus*	Natural	EY Lab.	Sia
2	WGA	*Triticum vulgaris*	Natural	EY Lab.	$(GlcNAc)_n$, polySia
3	PVL	*Psathyrella velutina*	Natural	Wako	Sia, GlcNAc
4	MAL	*Maackia amurensis*	Natural	Seikagaku	α2-3Sia
5	MAH	*Maackia amurensis*	Natural	Vector	α2-3Sia
6	ACG	*Agrocybe cylindracea*	Natural	JOM	α2-3Sia
7	rACG	*Agrocybe cylindracea*	*E.coli*	AIST	α2-3Sia
8	rGal8N	*Homo sapiens*	*E.coli*	AIST	α2-3Sia
9	SNA	*Sambucus nigra*	Natural	Seikagaku	α2-6Sia
10	SSA	*Sambucus sieboldiana*	Natural	Vector	α2-6Sia
11	TJAI	*Trichosanthes japonica*	Natural	Vector	α2-6Sia
12	rPSL1a	*Polyporus squamosus*	*E.coli*	AIST	α2-6Sia
13	ADA	*Allomyrina dichtoma*	Natural	JOM	α2-6Sia, Forssman, A, B
14	PHAL	*Phaseolus vulgaris*	Natural	Seikagaku	GlcNAcβ1-6Man (tetraantenna)
15	DSA	*Datura stramonium*	Natural	Seikagaku	GlcNAcβ1-6Man (tetraantenna)
16	TxLcI	*Tulipa gesneriana*	Natural	JOM	Galactosylated N-glycans up to triantenna
17	ECA	*Erythrina cristagalli*	Natural	Seikagaku	βGal
18	RCA120	*Ricinus communis*	Natural	Vector	βGal
19	rGal7	*Homo sapiens*	*E.coli*	AIST	Type1 LacNAc, chondroitin polymer
20	rGal9N	*Homo sapiens*	*E.coli*	AIST	GalNAcα1-4Gal (A), polyLacNAc
21	rGal9C	*Homo sapiens*	*E.coli*	AIST	PolyLacNAc, branched LacNAc
22	rC14	*Gallus gallus domesticus*	*E.coli*	AIST	Branched LacNAc
23	rDiscoidin II	*Dictyostelium dicodeum*	*E.coli*	AIST	LacNAc, Galβ1-3GalNAc (T), GalNAc (Tn)
24	BPL	*Bauhinia purpurea alba*	Natural	Vector	Galβ1-3GlcNAc(GalNAc), α/βGalNAc
25	rCGL2	*Homo sapiens*	*E.coli*	AIST	GalNAcα1-3Gal (A), polyLacNAc
26	PHAE	*Phaseolus vulgaris*	Natural	Vector	bisecting GlcNAc
27	GSLII	*Griffonia simplicifolia*	Natural	Vector	GlcNAcβ1-4Man
28	rSRL	*Sclerotium rolfsii*	*E.coli*	AIST	Core1,3, agalacto N-glycan
29	UDA	*Urtica dioica*	Natural	Vector	$(GlcNAc)_n$
30	PWM	*Phytolacca americana*	Natural	Vector	$(GlcNAc)_n$
31	rF17AG	*Escherichia coli*	*E.coli*	AIST	GlcNAc
32	rGRFT	*Griffithia sp.*	*E.coli*	AIST	Man
33	NPA	*Narcissus pseudonarcissu*	Natural	Seikagaku	Manα1-3Man
34	ConA	*Canavalia ensiformis*	Natural	Vector	M3, Manα1-2Manα1-3(Manα1-6)Man, GlcNAcβ1-2Manα1-3(Manα1-6)Man
35	GNA	*Galanthus nivalis*	Natural	Vector	Manα1-3Man, Manα1-6Man
36	HHL	*Hippeastrum hybrid*	Natural	Vector	Manα1-3Man, Manα1-6Man
37	ASA	*Allium sativum*	Natural	JOM	Galβ1-4GlcNAcβ1-2Man
38	DBAI	*Dioscorea batatas*	Natural	JOM	High-man
39	CCA	*Castanea crenata*	Natural	JOM	Galactosylated N-glycans up to triantenna
40	Heltuba	*Helianthus t uberosus*	Natural	JOM	Manα1-3Man
41	rHeltuba	*Helianthus tuberosus*	*E.coli*	AIST	Manα1-3Man
42	VVAII	*Vicia villosa*	Natural	JOM	Man, agalacto
43	rOrysata	*Oryza sativa*	*E.coli*	AIST	Manα1-3Man, highman, biantenna
44	rPALa	*Phlebodium aureum*	*E.coli*	AIST	Man5, biantenna
45	rBanana	*Musa acuminata*	*E.coli*	AIST	Manα1-2Manα1-3(6)Man
46	rCalsepa	*Calystegia sepium*	*E.coli*	AIST	Biantenna with bisecting GlcNAc
47	rRSL	*Ralstonia solanacearum*	*E.coli*	AIST	αMan, α1-2Fuc (H), α1-3Fuc (Lex), α1-4Fuc (Lea)
48	rBC2LA	*Burkholderia cenocepacia*	*E.coli*	AIST	αMan, high-man
49	AOL	*Aspergillus oryzae*	Natural	Vector	α1-2Fuc (H), α1-3Fuc (Le^x), α1-4Fuc (Le^a)
50	AAL	*Aleuria aurantia*	Natural	Vector	α1-2Fuc (H), α1-3Fuc (Le^x), α1-4Fuc (Le^a)
51	rAAL	*Aleuria aurantia*	*E.coli*	AIST	α1-2Fuc (H), α1-3Fuc (Le^x), α1-4Fuc (Le^a)
52	rPAIIL	*Pseudomonas aeruginosa*	*E.coli*	AIST	αMan, α1-2Fuc (H), α1-3Fuc (Le^x), α1-4Fuc (Le^a)
53	rRSIIL	*Ralstonia solanacearum*	*E.coli*	AIST	α1-2Fuc (H), α1-3Fuc (Le^x), α1-4Fuc (Le^a)
54	rPTL	*Pholiota terrestris*	*E.coli*	AIST	α1-6Fuc
55	PSA	*Pisum sativum*	Natural	Seikagaku	α1-6Fuc up to biantenna
56	LCA	*Lens culinaris*	Natural	Vector	α1-6Fuc up to biantenna
57	rAOL	*Aspergillus oryzae*	*E.coli*	AIST	α1-2Fuc (H), α1-3Fuc (Le^x), α1-4Fuc (Le^a)
58	rBC2LCN	*Burkholderia cenocepacia*	*E.coli*	AIST	Fucα1-2Galβ1-3GlcNAc/GalNAc
59	LTL	*Lotus tetragonolobus*	Natural	Seikagaku	Le^x, Le^y
60	UEAI	*Ulex europaeus*	Natural	Vector	α1-2Fuc
61	TJAII	*Trichosanthes japonica*	Natural	Vector	α1-2Fuc
62	MCA	*Momordica charantia*	Natural	JOM	α1-2Fuc
63	GSLI	*Griffonia simplicifolia*	Natural	Seikagaku	αGalNAc (A, Tn), aGal (B)
64	PTLI	*Psophocarpus tetragonolo*	Natural	Tokyo Kasei	αGalNAc (A, Tn)
65	GSLIA4	*Griffonia simplicifolia*	Natural	EY Lab.	αGalNAc (A, Tn)
66	rGC2	*Geodia cydonium*	*E.coli*	AIST	α1-2Fuc (H), αGalNAc (A), αGal (B)
67	GSLIB4	*Griffonia simplicifolia*	Natural	Vector	αGal (B)
68	rMOA	*Marasmius oreades*	*E.coli*	AIST	αGal (B)
69	EEL	*Euonymus europaeus*	Natural	Vector	αGal (B)
70	rPAIL	*Pseudomonas aeruginosa*	*E.coli*	AIST	α,βGal, αGalNAc (Tn)
71	LEL	*Lycopersicon esculentum*	Natural	Vector	Polylactosamine, $(GlcNAc)_n$
72	STL	*Solanum tuberosum*	Natural	Seikagaku	Polylactosamine, $(GlcNAc)_n$

73	rGal3C	*Homo sapiens*	*E.coli*	AIST	LacNAc, polylactosamine
74	rLSLN	*Laetiporus sulphureus*	*E.coli*	AIST	LacNAc, polylactosamine
75	rCGL3	*Coprinopsis cinerea*	*E.coli*	AIST	LacDiNAc
76	PNA	*Arachis hypogaea*	Natural	Vector	Galβ1-3GalNAc (T)
77	ACA	*Amaranthus caudatus*	Natural	Vector	Galβ1-3GalNAc (T)
78	HEA	*Hericium erinaceum*	Natural	JOM	Galβ1-3GalNAc (T)
79	ABA	*Agarics bisporus*	Natural	Vector	Galβ1-3GalNAc (T), GlcNAc
80	Jacalin	*Artocarpus integrifolia*	Natural	Seikagaku	Galβ1-3GalNAc (T), GalNAcα (Tn)
81	MPA	*Maclura pomifera*	Natural	Seikagaku	Galβ1-3GalNAc (T), GalNAcα (Tn)
82	HPA	*Helix pomatia*	Natural	Seikagaku	αGalNAc (A, Tn)
83	VVA	*Vicia villosa*	Natural	Vector	α,βGalNAc (A, Tn, LacDiNAc)
84	DBA	*Dolichos biflorus*	Natural	Vector	α,βGalNAc (A, Tn, LacDiNAc)
85	SBA	*Glycine max*	Natural	EY Lab.	α,βGalNAc (A, Tn, LacDiNAc)
86	rPPL	*Pleurocybella porrigens*	*E.coli*	AIST	α,βGalNAc (A, Tn, LacDiNAc)
87	rCNL	*Clitocybe nebularis*	*E.coli*	AIST	α,βGalNAc (A, Tn, LacDiNAc)
88	rXCL	*Xerocomus chrysenteron*	*E.coli*	AIST	Core1,3, agalacto N-glycan
89	VVA I	*Vicia villosa*	Natural	JOM	GalNAcβ1-3(4)Gal
90	WFA	*Wisteria floribunda*	Natural	Vector	Terminal GalNAc, LacDiNAc
91	rABA	*Agarics bisporus*	*E.coli*	AIST	Galβ1-3GalNAc (T), GlcNAc
92	rDiscoidin I	*Dictyostelium Discodeum*	*E.coli*	AIST	Gal
93	DBAIII	*Dioscorea batatas*	Natural	JOM	Maltose
94	rMalectin	*Homo sapiens*	*E.coli*	AIST	Glcα1-2Glc
95	CSA	*Oncorhynchus keta*	Natural	JOM	Rhamnose, Galα1-4Gal
96	FLAG-EW29Ch-E20K	*Lumbricus terrestris*	*E.coli*	AIST	6-sulfo-Gal

[1]Abbreviations: Gal (D-galactose), GalNAc (N-acetyl-galactosamine), GlcNAc (N-acetyl-glucosamine), Fuc (L-fucose), Glc (D-glucose), Sia (Sialic acid), LacNAc (N-acetyl-lactosamine).

[2]Specificity data was obtained by frontal affinity chromatography and glycoconjugate microarray.

[3]Abbreviations: JOM (J-OIL MILLS, INC), Vector (VECTOR LABORATORIES, INC), Seikagaku (SEIKAGAKU CORPORATION), EY (EY LABORATORIES, INC), AIST (National Institute of Advanced Industrial Science and Technology)

Supplemetary Table.3 Identification of rPALa-precipitated glycoproteins at 80 kDa by LC-MS/MS

Uniprot Accession number	Protein name	Molecular weight	Number of peptides[a]	Number of unique peptides[b]	Sequence Coverage (%)[c]	Spectral counting in HD[d]	Spectral counting in AP[d]
Q14624|ITIH4_HUMAN	Inter-alpha-trypsin inhibitor heavy chain H4	103357	12	12	14	2	15
P05106|ITB3_HUMAN	Integrin beta-3	87058	11	11	15	9	13
P25311|ZA2G_HUMAN	Zinc-alpha-2-glycoprotein	34259	7	7	26	0	8
P80188|NGAL_HUMAN	Neutrophil gelatinase-associated lipocalin	22588	2	2	12	0	2
P16671|CD36_HUMAN	Platelet glycoprotein 4	53053	4	4	8	0	4
P0C0L5|CO4B_HUMAN	Complement C4-B	192750	2	2	1	0	2
P0C0L4|CO4A_HUMAN	Complement C4-A	192784	2	2	1	0	2
P05120|PAI2_HUMAN	Plasminogen activator inhibitor 2	46596	2	2	5	0	2
P04114|APOB_HUMAN	Apolipoprotein B-100	515611	2	2	0	0	2
Q5T749|KPRP_HUMAN	Keratinocyte proline-rich protein	64136	2	2	2	0	2
P36952|SPB5_HUMAN	Serpin B5 OS=Homo sapiens	42100	2	2	6	0	2

[a]The number of identified peptides

bThe number of identified peptides, which contain specific sequence for each protein.

cThe ratio of the sequence of identified peptides to whole sequence of each protein.

[d]The number of spectrums which were used for identification of proteins

Supplementary Table 4. Spearman's rank correlation coefficient between the values of sandwich assays and ages in HDs and APs

		HD	AP
Tim4-αCD63	*p-value*	0.668	0.421
	Spearman's *ρ*	-0.083	-0.191
Tim4-αCD9	*p-value*	0.041	0.870
	Spearman's *ρ*	0.382	-0.039
Tim4-αCD41	*p-value*	0.564	0.830
	Spearman's *ρ*	0.112	0.051
Tim4-αCD61	*p-value*	0.107	0.582
	Spearman's *ρ*	0.306	0.131
Tim4-αCD81	*p-value*	0.046	0.813
	Spearman's *ρ*	0.374	0.057

Supplementary Table 5. Spearman's rank correlation coefficient among Tim4-based sandwich assays

	Tim4-αCD9	Tim4-αCD41	Tim4-αCD61	Tim4-αCD63	Tim4-αCD81
Tim4-αCD9	1.00[a]	0.82	0.79	0.61	0.53
Tim4-αCD41	0.82	1.00	0.91	0.69	0.26
Tim4-αCD61	0.79	0.91	1.00	0.63	0.24
Tim4-αCD63	0.61	0.69	0.63	1.00	0.33
Tim4-αCD81	0.53	0.26	0.24	0.33	1.00

Red : high correlation, *blue* : low correlation

[a]Spearman's ρ

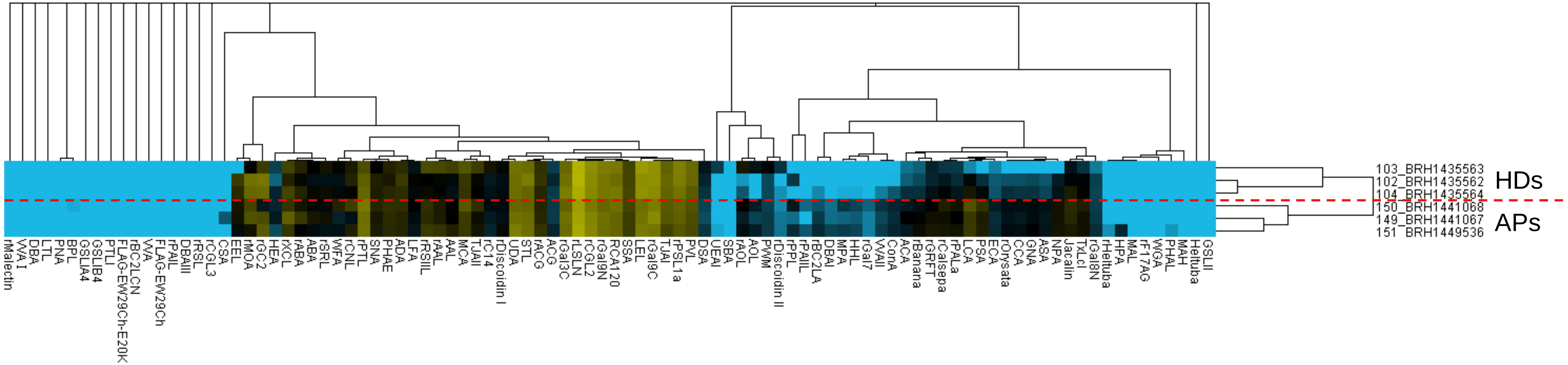

Supplementary Figure. 1 Cluster analysis of lectin microarray data of EVs purified from sera of HDs and APs.

Lectin microarray data of EVs purified from sera of APs (n = 3) and HDs (n = 3) were normalized, log-transformed, and analyzed by Cluster 3.0 with average linkage methods. The zero value of lectin signal was converted to 1. Sample No.4, 5, 6 for HDs and No.30, 31, 32 for APs were used in Supplementary Table 1. *yellow*: high, *blue*: low

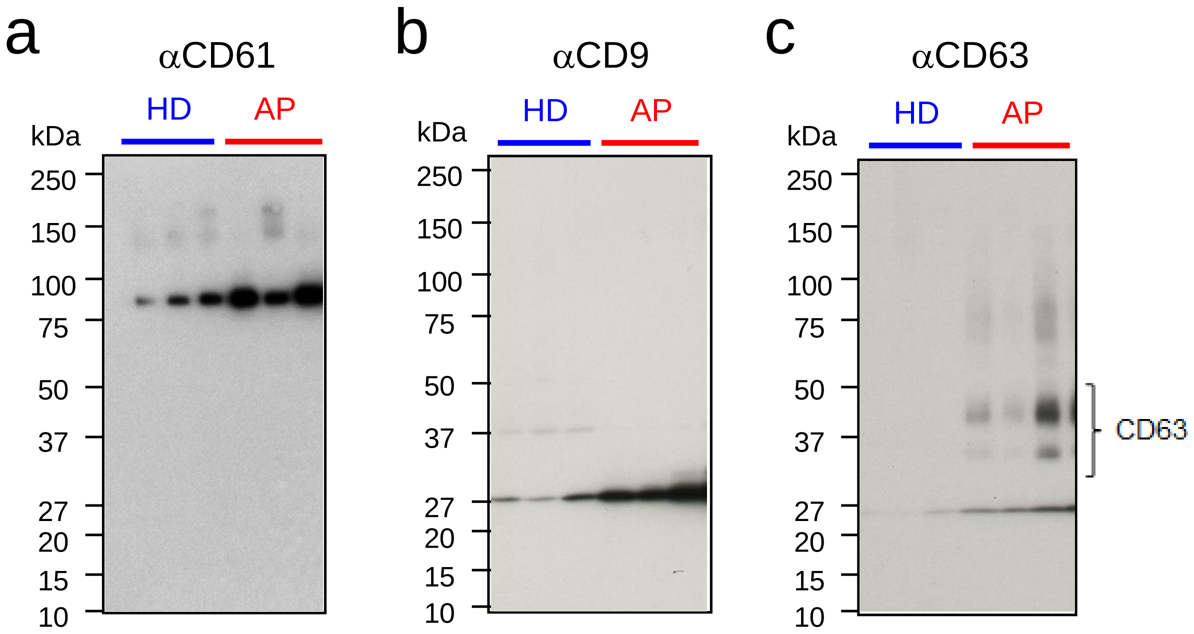

Supplementary Figure 2. Western blotting of HD- and AP-derived EVs. Equal amount of proteins (0.2 µg) of purified HD- and AP-derived EVs were separated on SDS-PAGE and blotted with αCD61 (a), αCD9 (b) and αCD63 (c). Lane 1: sample No. 4, lane 2: No. 5, lane 3: No. 6, lane 4: No. 30, lane 5: No. 31, lane 6: No. 32.

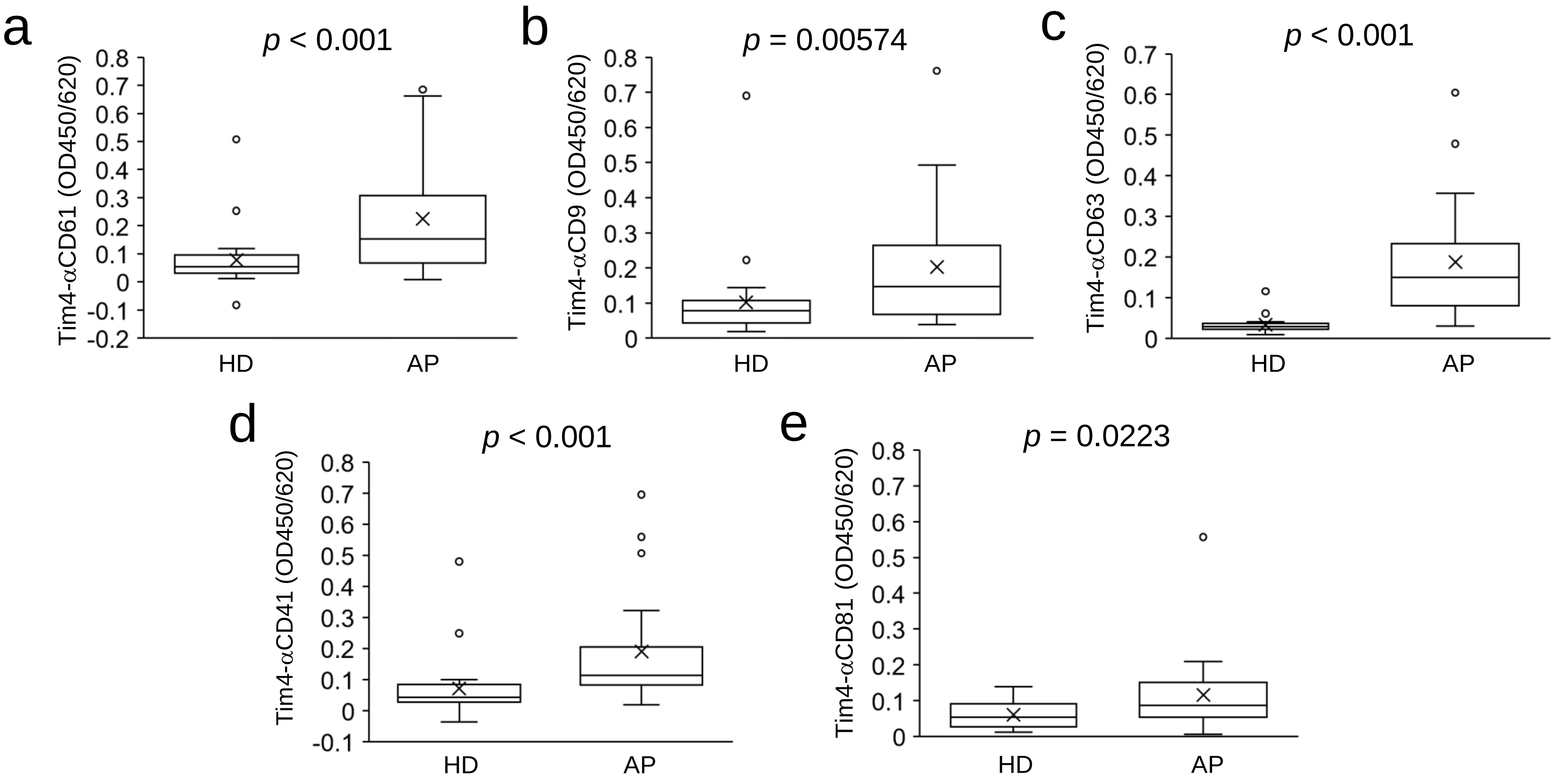

Supplementary Figure. 3 Quantitative analysis of HD- and AP-derived EVs using Tim4-based sandwich assays in whole cohort.

Box-whisker plots of the data of whole cohort (HDs: n = 29, APs: n = 20) analyzed by sandwich assays using immobilized Tim4 and overlay antibodies against CD61 (a), CD9 (b), CD63 (c), CD41 (d), and CD81 (e). OD: optical density. Sera used in this study are listed in Supplementary Table 1. *P*-values obtained by Wilcoxon-Mann-Whitney Test are indicated in the figure.

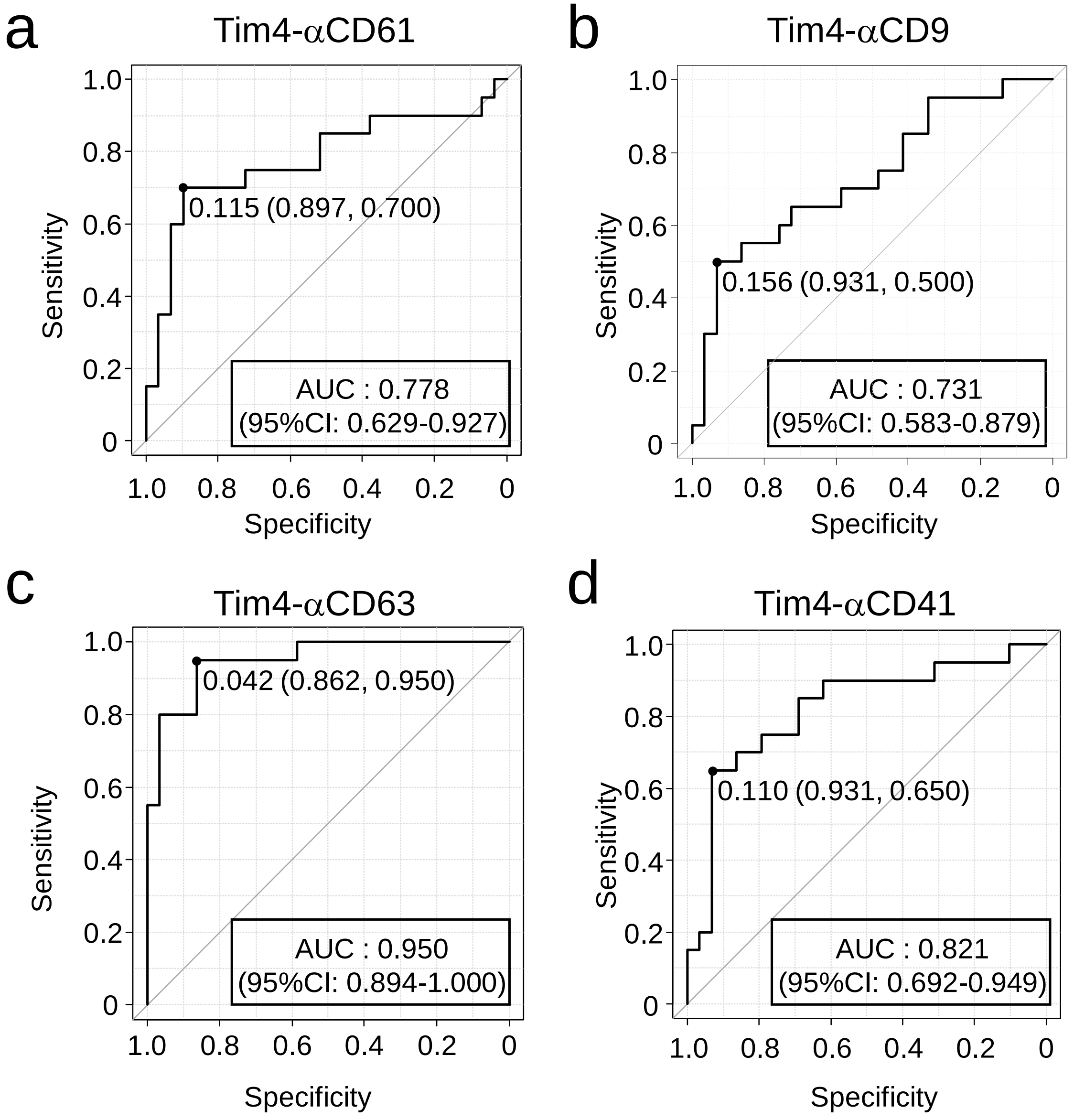

Supplementary Figure. 4 ROC curves of the values obtained by Tim4-based sandwich assays in whole cohort.

ROC curves for predicting AD risk by OD values obtained by sandwich assays (HDs: n = 29, APs: n = 20) using Tim4 and antibodies against CD61 (a), CD9 (b), CD63 (c), and CD41(d). Area-under-curve (AUC) and its 95% confidence interval (CI) are indicated in the figure.
